# Real-World Outcomes of Antiretroviral Therapy in People Living With HIV in Saudi Arabia: A Three-Year Observational Cohort Study From a Tertiary Care Center

**DOI:** 10.7759/cureus.99896

**Published:** 2025-12-22

**Authors:** Ali Alsaeed

**Affiliations:** 1 Infectious Disease Division, Department of Internal Medicine, Dammam Medical Complex, Dammam, SAU

**Keywords:** antiretroviral therapy, art, forgiveness period, hiv, hiv aids, human immuno-deficiency virus, saudi arabia, treatment adherence

## Abstract

Background: Treatment adherence is an essential determinant in the management of HIV. In an observational cohort study in Saudi Arabia, the clinical outcomes of antiretroviral therapy (ART) in terms of viral suppression and treatment adherence were evaluated among people living with HIV.

Method: In an open, single-center, observational cohort study, 700 patients were enrolled and followed for three years, either in person or virtually. The study aimed to help patients transition to the U = U (undetectable = untransmittable) category as per the Preventive Access Campaign. The viral load was estimated every three months. Treatment adherence was calculated based on the number of missed doses by the patients.

Results: In this observational cohort study, 700 patients received ART treatment of various types. The patients presented varying disease durations ranging from one to 20 years. The triple drug combination comprising bictegravir/emtricitabine/tenofovir (B/F/TAF) was the most prescribed ART. At the end of 36 months, 616 patients reached the U = U category. A significant reduction in viral load was observed at 12, 24, and 36 months from baseline (p < 0.05; 95% confidence interval (CI)). All patients tolerated the ART, and there were no treatment discontinuations due to side effects. The average treatment adherence was more than 95% across all the cohorts. An increase in viral load was observed with missing doses; however, the newer ARTs effectively controlled viral load during the ART-reduced adherence.

Conclusions: HIV is treatable, and with effective treatment, patients can achieve “untransmittable” status. This study demonstrates the effectiveness of the newer ARTs in HIV management in Saudi Arabia.

## Introduction

The HIV infection has affected millions of people worldwide. The Joint United Nations Program on HIV/AIDS (UNAIDS), 2022, reports that approximately 39 million people were living with HIV globally, of which an estimated 25.6 million were in sub-Saharan Africa, accounting for nearly two-thirds of all HIV infections in the globe [1-3]. An estimated 1.3 million individuals worldwide acquired HIV in 2023, marking a 39% decline in new HIV infections since 2010 and a 60% decline since the peak in 1995 [4,5]. It must be noted that the early diagnosis of HIV infection and effective long-term use of antiretroviral therapy (ART) are resulting in a decline in the cases. The availability and accessibility of combination antiretroviral treatment are increasing and progressing toward disease control [6].

Despite current estimates indicating that Middle Eastern countries constitute only a proportion of the 36.9 million infected with HIV worldwide, the number of new infections in the Middle East and North Africa (MENA) region has increased by 26% since 2015 [7]. HIV infection is a notifiable disease, and almost all countries have implemented national programs for HIV control [5].

In the Kingdom of Saudi Arabia, the national control programs have improved HIV surveillance with advances in medical care, counseling, family planning, diagnostic evaluation, and ART [7]. However, the exact incidence and clinical evidence of ART in Saudi Arabia have not yet been reported [8]. ART for HIV is lifelong [9]. Therefore, choosing an effective ART combination and treatment adherence are essential parameters to maintain viral suppression [10]. Being a lifelong treatment, adherence to the daily medication is the most critical factor in disease control and being untransmittable in the community. Although treatment adherence in HIV is greatly emphasized to the patients, missing doses are occasionally observed and reported [11]. Missing doses for a prolonged period reactivates the virus, leading to an "active infection" status for the patient [12]. In addition to virus reactivation, the emergence of resistant strains poses further challenges for treatment [13,14]. Thus, an effective treatment algorithm emphasizes patient education for treatment adherence throughout their life for HIV control.

An observational cohort study was planned to evaluate the efficacy of various ARTs in patients living with HIV (PLHIV) in Saudi Arabia. This paper presents the findings of this observational cohort study.

## Materials and methods

An open, prospective, non-comparative, observational cohort study was planned in PLHIV in Saudi Arabia with the following study objectives.

Primary objectives

The primary objectives include evaluating ART efficacy in terms of viral suppression and clinical outcomes over a three-year follow-up period in PLHIV in Saudi Arabia and assessing the impact of treatment adherence on viral suppression during the study period among PLHIV in Saudi Arabia.

Secondary objective

The secondary objective is to assess safety and tolerance to ART among the participants.

Adult HIV-positive confirmed cases receiving ART treatment above 18 years of age, of both sexes, visiting the institute were the study participants. Participant inclusion was subject to their understanding and agreement to provide informed consent. During 2022-2023, clinical management at our center followed the Saudi HIV Guidelines, Second Edition, which were the nationally adopted reference at that time. The Third Edition (2024) was implemented thereafter [15]. Baseline blood samples were collected for estimating the HIV viral load. Female patients of reproductive age were counselled on contraception. All women of reproductive age were asked to promptly report pregnancy to the treating clinician during the study period. Participants with other comorbidities, such as tuberculosis and hepatitis B or C, were eligible. All participants received a diary to accurately and consistently record their daily medication intake. Participants were advised not to take a double dose to make up for the dose missed the previous day. At each follow-up visit, participants were asked to bring the patient diary for the investigator's review.

Participants were followed up every three months (four visits per year) over the three-year study period. Participants were expected to visit in person and/or virtually, as scheduled. Hybrid consultations (in-person and virtual) were permitted. If the participant was unable to visit on the planned date, they were reminded by a telephone call from the center. At each visit, the participant underwent a clinical examination and laboratory tests to assess CD4 count and viral load. Based on the patient's general health status and the estimated viral load, the study investigator was free to modify the participant's ART. At each follow-up visit, the investigator asked the participant to report any adverse events and to indicate whether any doses had been missed since the last follow-up visit. All data for the participant were captured in the patient's hospital source data file. At each visit, participants were advised not to miss any doses and to report promptly to the study investigator any side effects or adverse reactions they experienced.

Sample size and biostatistics

The study, being an observational cohort, considered a sample size of 700 subjects as the representative sample size for the institute dedicated to HIV care in the country. No special sample size estimations were used, and this sample size may be considered a convenience sample. The collected data were captured in Excel format (Microsoft Corp., Redmond, WA, US) for statistical analysis. The statistical analysis included collation of the demographics data, such as age, sex, history of HIV infections, duration of the disease, treatment received at diagnosis, and current therapy, including viral load at diagnosis if available in the patient records. The statistical significance of the difference between viral load at study enrollment and CD4 count at follow-up visits was assessed using the paired Student t-test for the entire cohort, for subjects who visited in person, and for those who had hybrid consultations. A chi-squared test was used to assess the significance of differences in average missing doses across the three years. Statistical significance was considered when the p-value was <0.05 at a 95% confidence interval (CI). The correlation between missing doses and their effect on viral load was calculated. Microsoft Excel was used for all the analysis.

## Results

The study was conducted over three calendar years, from January 2022 to December 2024, enrolling 700 participants. Before its initiation, the study protocol was approved by the Institutional Review Board. Informed consent was obtained from all participants before enrollment. The cohort consisted of 571 male participants (510 Saudi nationals) and 129 female participants (122 Saudi nationals), for a total of 226 males and 345 females. Among the male participants, 226 were single, and 345 were married. Among the female participants, 88 were married, and 41 were single. The average age among female participants was 39 (±14.4) years, and the average age among male participants was 41 (±13.7) years. Among the comorbidities, psychiatric issues were the highest reported in 129 patients, followed by a history of drug use in 90 patients and tuberculosis in 70 patients. Hepatitis B and hepatitis C were reported in 31 and eight patients, respectively. Out of 700 participants, 50 participants opted for “in-person” consultations at all visits, and 650 participants opted for the hybrid mode.

**Figure 1:**
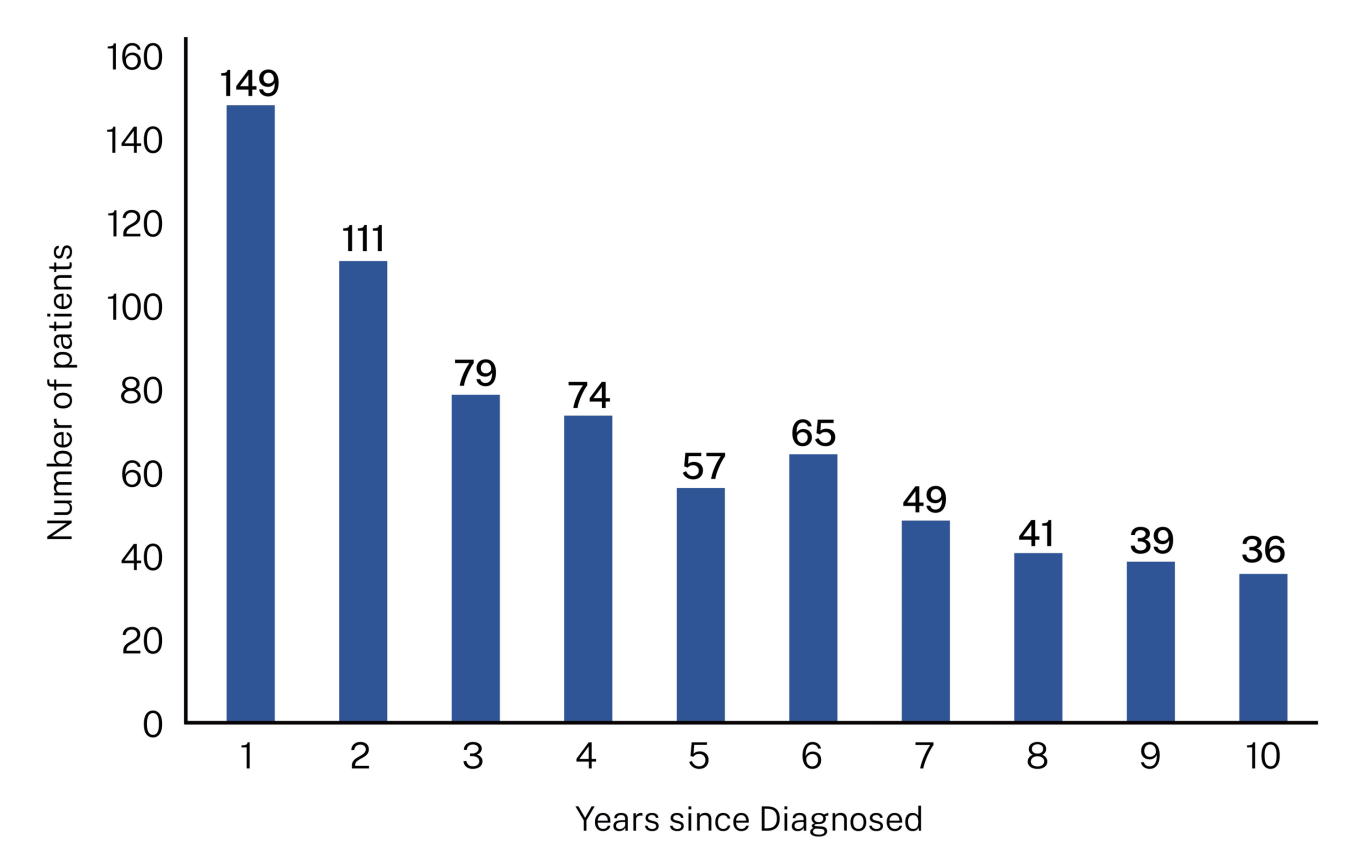
Duration of HIV among study participants The study population included patients diagnosed with HIV one year ago, as well as those diagnosed 10 years ago.

Among the 700 participants, the average duration of the disease was 7.95 (±5.65) years; there were participants diagnosed one year before enrollment, as well as those with a history of disease lasting 20 years (Figure 1: distribution of disease duration in the cohort).

**Figure 2:**
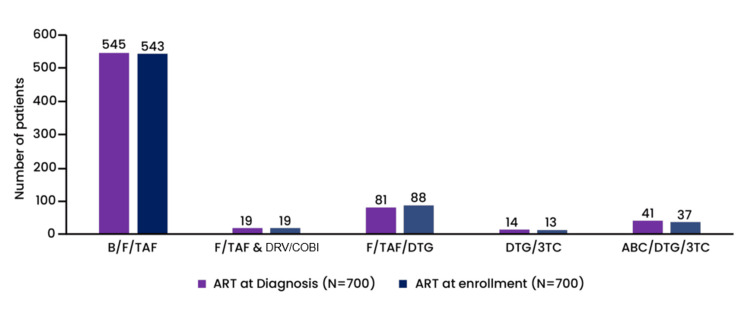
ART at diagnosis and at enrollment The ART treatment received by the patient at diagnosis and currently being taken at enrollment is depicted in the figure. B/F/TAF: bictegravir/emtricitabine/tenofovir; F/TAF & DRV/COBI: emtricitabine/tenofovir and darunavir/cobicistat; F/TAF/DTG: emtricitabine/tenofovir/dolutegravir; DTG/3TC: dolutegravir/lamivudine; ABC/DTG/3TC: abacavir/dolutegravir/lamivudine; ART: antiretroviral therapy

The combination product bictegravir 50 mg/emtricitabine 200 mg/tenofovir alafenamide 25 mg (B/F/TAF), administered once daily (trade name Biktarvy), was the most prescribed at diagnosis among patients (545/700). At enrollment, participants' general health status was excellent in 23.57%, very good in 24.57%, good in 29.85%, fair in 16.71%, and poor in 5.28%. The study investigator considered changing ART in patients based on laboratory test results and the participants' overall health status. Figure 2 illustrates the ART treatment prescribed to the patients at diagnosis and at the time of study enrollment.

At enrollment, 445 patients had viral loads below undetectable levels; the average viral load detected in the entire cohort was 197,946.63 (±136,353.57), which decreased to 65.13 (±192.59) at month 36 (p-value < 0.05, 95% CI). Table 1 presents the reduction in viral load from baseline across the entire cohort; a similar decrease was observed among patients who opted for hybrid or in-person consultations. A statistically significant decrease in viral load (p-value < 0.05) was observed in the entire cohort compared to enrollment.

**Table 1:** Evolution of VL from enrollment to the end of study Statistically significant reduction in VL observed in the entire cohort at the end of 36 months. ART was effective in reducing VL among all PLHIV. *Significant VL reduction from enrollment (p < 0.05); Student t-test VL: viral load; PLHIV: patients living with HIV; ART: antiretroviral therapy

	HIV VL at enrollment (entire cohort) n = 700	VL at 36 months (entire cohort) n = 700	VL at 36 months (hybrid consultations) n = 650	VL at 36 months (in-person consultations) n = 50
Average	197,946.63	65.1314285714286*	66.5753846153846*	46.36*
SD	136,353.57	192.5972541	194.0024653	173.977451
Min	25	0	0	0
Max	397,632.00	987	987	888

The CD4 counts at three months and at 36 months in the entire cohort are presented in Table 2. In the hybrid consultation group, the CD4 count at three months was 647.51 (± 398.69), and at 36 months, it was 663.16 (± 399.86). In the in-person consultation group, the CD4 count at three months was 665.05 (± 399.24), and at 36 months, it was 676.38 (±394.46). The change in CD4 count at 36 months from three months was not statistically significant (p-value > 0.05).

**Table 2:** Change in CD count-entire cohort There was no statistical difference in CD4 count between 3 months and 36 months in the entire study cohort.

	CD4 @ month 3	CD4 @ month 36	
Average	648.7454874	664.1085714	p-value > 0.05; not significant; Student t-test
SD	398.3988095	399.2143747	
Min	63	60	
Max	1,430	1,425	
NA	146	0	

**Figure 3:**
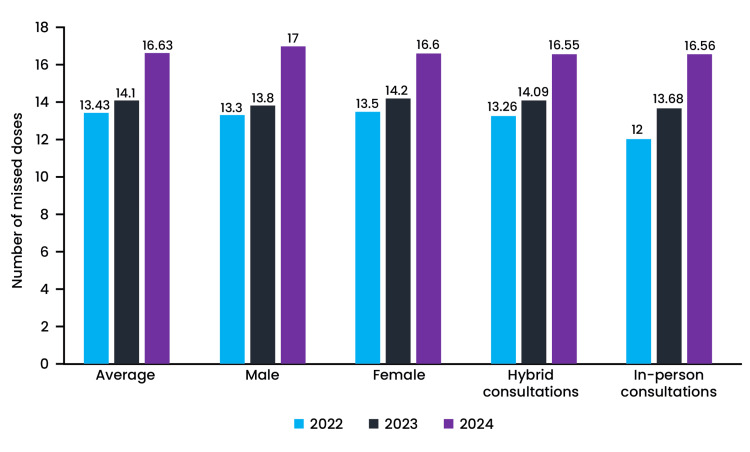
Average doses missed by the subjects during the 36-month study period No statistically significant difference was observed between the average missed doses, missed doses between male and female participants, and missed doses between in-person and hybrid consultations.

Seventy patients (10%) reported side effects over the three years; these were mild and did not necessitate any medical emergencies. All side effects resolved and did not lead to any treatment discontinuation and/or withdrawal of the participants from the study. All participants had at least one missed dose during the three-year study period (Figure 3).

**Figure 4:**
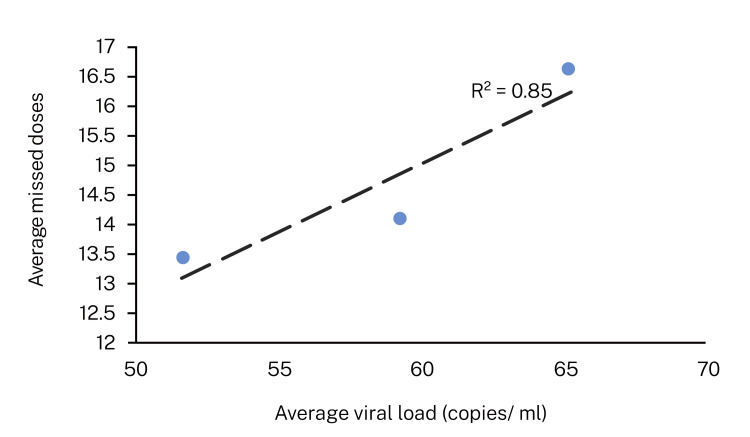
Correlation between missing doses and viral load at 12, 24, and 36 months Missing doses were associated with an increase in viral load. In year 3, the incidence of missing doses was higher than in the preceding two years among the study participants.

Hybrid and in-person group patients missed the most doses in year 3 compared with years 1 and 2. The hybrid group had missed 16.6 (±8.3) doses, and the in-person group had missed 17 (±7.38) doses in year 3, respectively. There was no statistical difference between the average doses missed by the groups over three years. No statistically significant difference was observed between male and female participants in terms of missed doses over the three-year study period. Between in-person and hybrid consultations, there was no statistical difference in the number of missed doses. The average viral load in the hybrid consultation group was 540.92 (±220.77) and 579.5 (±248.65) in the in-person consultation group at the end of 36 months. At the beginning of the study, 445 participants had an undetected viral load at enrollment. During the three years, 37 patients in year 1, 52 patients in year 2, and 58 patients in year 3 had detectable viral loads due to missed doses. Correlation studies demonstrated that missed doses are associated with higher viral load (Figure 4).

**Figure 5:**
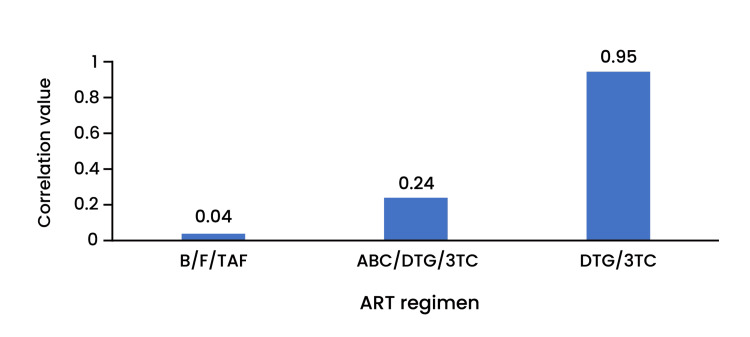
Correlation between missing doses of the ART regimen and viral load ART regimen B/F/TAF demonstrated the lowest correlation when doses were missed, resulting in the lowest increase in viral load during the forgiveness period. B/F/TAF: bictegravir/emtricitabine/tenofovir; ABC/DTG/3TC: abacavir/dolutegravir/lamivudine; DTG/3TC: dolutegravir/lamivudine; ART: antiretroviral therapy

Correlation studies on viral suppression and the intervention showed that, for a few brands, despite missed doses, viral suppression was sustained (Figure 5). The triple combination therapy of B/F/TAF demonstrated a correlation value of 0.04, indicating only a minimal increase in viral load during reduced ART adherence compared to the combination of abacavir, dolutegravir, and lamivudine (ABC/DTG/3TC) and dolutegravir with lamivudine (DTG/3TC). These results underscore the superior adherence forgiveness of B/F/TAF, making it a robust option for maintaining viral suppression even in the context of imperfect adherence.

**Figure 6:**
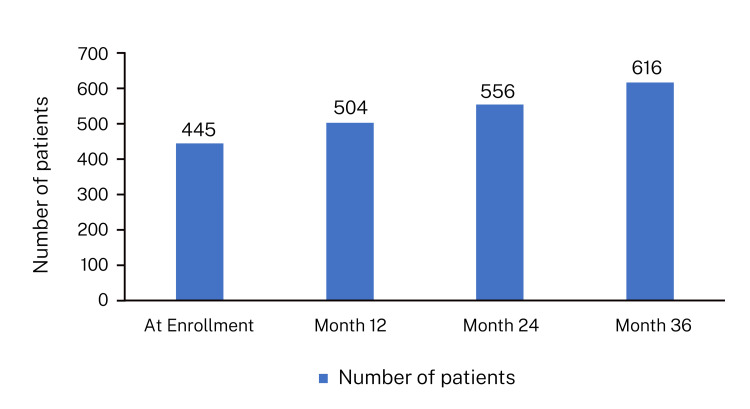
Number of patients with undetectable viral load at 12, 24, and 36 months ART was effective in reducing viral load in patients to undetectable levels, bringing 88% patients in the U = U category.

In the entire cohort, at the end of year 3, 616 (88%) patients achieved a viral load of zero, transitioning to the “untransmittable” category (Figure 6: patients showing undetectable viral load).

Viral suppression was observed with all interventions across the entire cohort, irrespective of gender, age, or disease duration.

## Discussion

The choice of an effective ART combination and regularity in medication have helped reduce HIV globally [16]. This study demonstrates the effectiveness of the ART regimen in the patient cohort. In-person and hybrid consultations were effective, and clinical care was delivered efficiently to participants.

The study group was diverse in terms of disease duration and treatment at enrollment. Newly diagnosed patients with one year of disease history, as well as those with 20 years or more of disease history, were enrolled in the cohort. Four hundred forty-five patients had undetectable viral loads, indicating the effectiveness of the current treatment. Based on the disease history, current viral load, and health status, the ongoing treatment was either continued or changed. The single-dose combination tablet containing B/F/TAF was the most commonly prescribed treatment option at enrollment and throughout the treatment period in this study. The combination treatment in HIV is very important as different ARTs have different mechanisms of action by which they attach themselves to the HIV, preventing replication. Currently, the HIV treatment comprises five distinct classes of ARTs that target multiple steps of the HIV life cycle (Table 3).

**Table 3:** Antiretroviral drug classes and examples [17,18]

Nucleoside reverse transcriptase inhibitors (NRTIs)	Abacavir, emtricitabine, lamivudine, tenofovir disoproxil fumarate, zidovudine
Non-nucleoside reverse transcriptase inhibitors (NNRTIs)	Efavirenz, etravirine, nevirapine, rilpivirine
Fusion inhibitors (FIs)	Enfuvirtide
Protease inhibitors (PIs)	Atazanavir, darunavir, fosamprenavir, ritonavir, saquinavir, tipranavir
Chemokine receptor 5 (CCR5) antagonist	Maraviroc
Integrase strand transfer inhibitors (INSTIs/IIs)	Dolutegravir, raltegravir, elvitegravir (given only in combination), bictegravir (given only in combination)
Post-attachment inhibitors	Ibalizumab
Pharmacokinetic enhancers	Cobicistat

The availability of combined ART has helped in suppressing HIV viremia, restoring the immune system, and improving the quality of life of HIV-infected patients [19]. Dealing with communicable diseases poses the challenge of the emergence of resistant strains, and this challenge is not new to HIV infections [20]. The emergence of drug-resistant and multidrug-resistant strains remains an important contributor to the current ART failure, associated with a higher risk of HIV disease progression and mortality [21]. Cross-resistance between drugs within the same class is known for all major ART drugs and is one of the reasons for treatment failure [22,23]. In this study, all patients responded to the therapy, and an increasing number remained non-transmitters at the end of the 36-month study period. Six hundred sixteen participants from the 700 enrolled were untransmittable, demonstrating precise choice of treatment intervention and its effectiveness. Ten percent of patients reported mild side effects that resolved within a reasonable time. None of the side effects necessitated treatment withdrawal or a change in ART. The single-dose combination tablet containing B/F/TAF (trade name Biktarvy^®^), emtricitabine/tenofovir alafenamide (F/TAF) (trade name Descovy^®^) with dolutegravir (DTG) (trade name Tivicay^®^), ABC/DTG/3TC (trade name Trimaq^®^), and DTG/3TC (trade name Dovato^®^) were the prescribed interventions in descending order in the study. The ART treatment was well tolerated by the entire cohort, and there were no withdrawals due to any treatment side effects.

Treatment adherence in HIV is critical, and patients should not miss doses [24]. Therefore, during every consultation, the importance of regular medication intake was emphasized to both the patient and their caregivers. However, instances of missing doses were reported. Each patient missed at least one dose during the study period. In years 1 and 2, the average number of doses missed was lower than that in year 3, possibly because patients were becoming somewhat less diligent in their treatment adherence as viral load diminished. There was no statistically significant difference in viral load between the two consultation groups.

To ensure long-term success in HIV infection, the ability to control HIV replication beyond the dosing interval because of imperfect adherence is a crucial factor defined as forgiveness [25-28]. When applied to ART, it refers to the ability of a given regimen to achieve and maintain complete viral suppression despite a documented imperfect medication adherence [26,27]. In the real world, substantial clinical and therapeutic decisions are made based on this concept, although no established quantitative measure exists [29].

The correlation results between doses missed and viral load showed that the combination treatment with B/F/TAF maintained the lowest viral load during the ART forgiveness compared to other interventions. These three ART agents (B/F/TAF) have different mechanisms of action, each attaching to a different target in the causative organism. Thus, it exerts a potent antiretroviral action, leading to viral suppression. All these agents also have long half-lives, thereby maintaining effective inhibitory therapeutic concentrations in the blood. Therefore, missing a dose does not result in an immediate massive surge in the viral load. In cases of imperfect adherence, the intervention can control viral replication. However, persistent missed doses lead to viral reactivation, increasing viral load [30-32].

In the treatment of HIV, reported adherence levels greater than 95% have been able to maintain virologic suppression [33,34]. However, in real-world settings, lower adherence rates have been reported. Most studies indicate that 40%-60% of patients are less than 90% adherent, and adherence tends to decrease over time [13,35,36]. In this study, average treatment adherence was ~96%, and 88% of patients had undetectable viral load at the end of the study. All patients had significantly lower viral load at the end of 36 months. However, despite the set parameter U = U, 12% of patients remained outside this parameter, presenting lower viral loads. The quality of consultation, investment in patient education, awareness, and follow-up, along with the selection of effective interventions, have been critical factors in achieving high levels of treatment adherence and clinical outcomes among Saudi patients. This study is probably the first to examine treatment efficacy and adherence among PLHIV patients in Saudi Arabia.

The combination of B/F/TAF was considered as the first-line therapy. This combination is regarded as highly potent and has a high genetic barrier to resistance. Its effectiveness during ART forgiveness has also been reported [37,38]. Many other studies have also indicated a moderate forgiveness by other ART interventions. Successive studies have shown that moderate deviations from perfect adherence to ART drugs have been sufficient to control viral replication during the initial period [39-41].

Various researchers have used different HIV-RNA cut-offs to define virological success. From both clinical and laboratory perspectives, the definition of an undetectable viral load has been variable. HIV-RNA < 200 copies/mL has been considered as the value that prevents sexual HIV transmission, and that excludes low-level viral blips from failures [42]. Since then, randomized controlled trials and observational studies have confirmed that there have been no HIV sexual transmissions that occur from a person with a durably suppressed viral load of <200 copies/mL to a sexual partner [43-45]. In 2016, the Preventive Access Campaign launched the U = U initiative, “Undetectable equals untransmittable,” which states that a person living with HIV who has an undetectable plasma HIV RNA viral load cannot pass HIV to another person through sexual contact [46]. Today in the United States, viral load in plasma of <20 copies/mL, measured by the nucleic acid amplification technology PCR, is considered untransmittable [42].

Over the three years of treatment, the study has been reasonably successful, converting 616 of 700 patients to the undetectable category. The ART treatment was well tolerated, and treatment safety was demonstrated. The results can be generalized to other clinical settings in Saudi Arabia. The government of Saudi Arabia launched a nationwide HIV control program with an aim for prevention and effective treatment; the institute, being a dedicated HIV center, achieved these results, which testify that reasonable control in HIV treatment was achieved. Additionally, transmission was prevented, with more than 80% of patients achieving undetectable viral load. The study achieved an almost-zero viral load despite a few issues with treatment adherence. Adherence remains a crucial determinant, and non-adherence is a universal phenomenon strongly linked to human psychology and behavioral theory. Despite instances of non-adherence, the study has achieved viral suppression in the majority of patients. Although the study period is officially closed, participants are being followed up at the clinic according to the treatment and follow-up protocol in place at the center. As of the current date, all patients are alive and in good health, and the ART is ongoing. It is planned to use digital technology to enhance treatment adherence and establish patient support groups to improve treatment outcomes among Saudi PLHIV patients. This treatment and follow-up model can also be considered within Saudi Arabia's national health policy.

Limitations and strengths of the study

These results are from a single center, which is a limitation of the project. However, a sample size of 700 is representative and realistic for real-world clinical experience. The history of the disease and the treatments received were highly diverse in the subject population, and sizable proportions of study participants had undetectable viral load at enrollment. In such a situation, accurately evaluating the intervention's effect on viral load reduction is difficult.

We could have used digital technology to monitor patients' daily treatment adherence, recording the exact date and time of missed doses; this is another limitation we plan to address in our next project. Unlike many other studies, no cut-off threshold was established in this study; the study aimed to convert patients to an undetectable level, and we believe that ART was effective in maintaining undetectable levels among those subjects who were regular in their medicine intake, reemphasizing the importance of treatment adherence, and this can be considered a strength of this study.

## Conclusions

Taking each dose of ART, every day, has long been considered essential for reducing the risk of virological failure, development of drug resistance, and HIV transmission. Modern ART medications have long half-lives and lower resistance rates and are more forgiving of missed doses than previous generations of ART. These properties are helping with virological control and converting seropositive patients to the U = U category. This study demonstrates effective use of intervention in treating HIV along with health advocacy, treatment safety, and satisfactory clinical outcomes in real-world clinical settings.
